# Verteilungspolitische Herausforderungen der Corona-Krise

**DOI:** 10.1007/s10273-021-2816-6

**Published:** 2021-01-19

**Authors:** Überblick

**Keywords:** D31, E62, H12

## Abstract

Die Corona-Krise hat sich insbesondere auch auf den Arbeitsmarkt ausgewirkt. Der Lockdown hat zu Arbeitsplatzverlusten geführt - allen voran in der Hotel-, Gastronomie- und Tourismusbranche. Ebenso sind bestimmte Branchen von Kurzarbeit betroffen. Das hat Folgen für die Einkommenssituation der betroffenen Haushalte. Es lässt sich zeigen, dass Haushalte mit bereits vor der Krise niedrigeren Einkommen in der Krise stärkere Einkommensverluste erleiden. Allerdings entfaltet das Konjunktur- und Krisenbewältigungspaket der Bundesregierung mit Hilfsmaßnahmen durchaus seine Wirkung, um Arbeitsplätze zu stabilisieren und soziale Härten abzufedern. Davon profi tieren Männer jedoch vergleichsweise stärker als Frauen.

## Abstract (en)

The Coronavirus crisis has had a distinct impact on the labour market. The lockdown has led to job losses, especially in the catering and tourism industries. Certain sectors have also been affected by shortterm work. This also has consequences for the income situation of affected households. It is evident that households with incomes that were already lower before the crisis are more affected by income losses during the crisis. However, the federal government’s economic stimulus and crisis management aid package is certainly effective at stabilising jobs and averting social hardship. However, men benefit from this comparatively more than women.

